# Evaluating the Short-Term Impact of Closed Versus Open Reduction Techniques on Acetabular Remodeling in Developmental Hip Dysplasia

**DOI:** 10.7759/cureus.78997

**Published:** 2025-02-14

**Authors:** Khalid Bakarman, Abdurahman K Addweesh, Aljoharah I Albnyan, Munib N Alkhateb, Zulqurnain Rafiq

**Affiliations:** 1 Pediatric Orthopedics, King Saud University, Riyadh, SAU; 2 College of Medicine, King Saud University, Riyadh, SAU; 3 Orthopedics, King Saud Medical City, Riyadh, SAU

**Keywords:** acetabular development, acetabular index, avascular necrosis, developmental dysplasia of the hip, open reduction

## Abstract

Introduction: Developmental dysplasia of the hip (DDH) encompasses abnormalities in the acetabulum or the femoral head, ranging from mild dysplasia to complete dislocation. The primary goal of DDH treatment is to achieve and maintain a concentric reduction of the hip joint, allowing for normal acetabular and femoral head remodeling.

Method: This retrospective study reviewed records of patients treated for idiopathic DDH between 2016 and 2021 at a single institution. Patients included had undergone either closed or open reduction and completed at least two years of follow-up. Demographic data and radiographic assessments at multiple intervals postreduction were collected and analyzed using IBM SPSS Statistics for Windows, Version 29 (Released 2023; IBM Corp., Armonk, New York, United States).

Results: A total of 41 patients (64 hips) were included, with a mean age at reduction of 6.92 months and a follow-up duration of 51.5 months. Closed reduction was performed on 38 hips, while 26 hips underwent open reduction. The mean age at reduction was significantly lower in the closed reduction group (4.58 months) compared to the open reduction group (10.34 months). The mean preoperative acetabular index (AI) decreased from 37.39 to 19.54 in the closed reduction group and from 36.74 to 19.4 in the open reduction group.

Conclusion: The study found no significant difference between closed and open reduction techniques in acetabular remodeling. While residual dysplasia and avascular necrosis (AVN) were more common in the closed group, the differences were not statistically significant. Further research with larger sample sizes and longer follow-up periods is recommended to confirm these findings.

## Introduction

Developmental dysplasia of the hip (DDH) refers to abnormalities in the size, shape, or orientation of the acetabulum or the femoral head. DDH includes a spectrum of conditions that range from mild acetabular dysplasia to complete dislocation of the femoral head from the acetabulum. In most cases, abnormality arises due to the maldevelopment of the acetabulum [1]. Femoral head involvement can be secondary due to nonphysiological biomechanics that can be caused by anteverted acetabulum. The hip becomes unstable due to the loss of tight-fitting between the femoral head and the acetabulum. Consequently, the femoral head starts to move over outside the confines of the acetabulum [2]. DDH can lead to significant morbidity if not diagnosed and treated promptly. The global prevalence of DDH varies from 0.1 to 6.6 cases per 1000 births [3]. Furthermore, it is a causative factor for approximately 30% of total hip arthroplasty procedures in individuals aged above 60 years [3]. The incidence rate, however, varies in different regions. These variations can be due to epigenetic factors or consanguinity incidence among different populations [3]. The condition is more prevalent in females, first-born children, and those with a positive family history [4]. The incidence of DDH is approximately sevenfold in siblings in families with a history of DDH compared to the general population [5]. The exact etiology of DDH remains unknown; however, several risk factors have been identified. A meta-analysis of 30 studies reported that breech presentation, family history, clicking hips, and female gender are at increased risk of DDH [6].

The main goal of treatment in DDH is to achieve and maintain a concentric reduction of the hip joint to allow for normal acetabular and femoral head remodeling, which will ultimately lead to the resolution of acetabular under coverage. The treatment, however, varies with the age of the patient at the time of presentation [7]. If treatment fails, residual dysplasia leads to an increase in loading across the joint at maturity which can be a significant risk for osteoarthritis. There is evidence suggesting that the acetabulum's ability to undergo remodeling significantly decreases after the age of four years, highlighting the importance of early detection and intervention [8]. The treatment options for DDH include Pavlik harness, closed reduction, open reduction, and osteotomies. Pavlik harness is generally used in infants younger than six months. However, higher failure rates are reported with Pavlik harness when the infant reaches sitting age. After six months, older infants and toddlers may require more invasive interventions like closed or open reduction [9]. Closed reduction treatment is used in infants who fail to respond to the Pavlik harness. This procedure involves the manual repositioning of the dislocated femoral head into the acetabulum followed by immobilization with a spica cast for three months. The quality of reduction following the procedure is assessed by arthrography with fluoroscopy. Closed reduction is not always successful in older children or those with severe dysplasia [10].

Open reduction is typically recommended for patients aged above 18 months. However, it is used for all children who fail the closed reduction procedure. Some studies suggest that diagnosis of DDH after three months of age is a late diagnosis. The open reduction procedure is 12 times more likely to be required in children aged ten months and above compared to those diagnosed before six weeks of age [11]. It involves surgically opening the hip joint which allows direct visualization and correction of anatomical abnormalities. In some cases, open reduction is combined with additional procedures like femoral or pelvic osteotomies. Yilar et al., in their study, reported that open reduction with Pemberton osteotomy resulted in better acetabular indices and revision surgery compared to open reduction only [12]. Successful acetabular remodeling is crucial to achieve a stable hip joint. Despite treatment, complications like residual dysplasia can still occur in which the acetabulum remains shallow or dysplastic. Residual dysplasia can predispose patients to early hip osteoarthritis and other complications later in life [13]. The decision between closed and open reduction is influenced by several factors, including the child's age, the severity of the dysplasia, the stability of the hip joint, and the presence of ossific nuclei. Closed reduction is typically performed in younger children due to its less invasive approach. However, open reduction is generally recommended when closed reduction is unsuccessful, when the patient is older at the time of presentation, or in cases involving fixed or teratologic dislocation. Currently, there is no consensus regarding the outcomes between open and closed reduction procedures.

Therefore, this study was conducted to assess the effects of both closed and open reduction techniques on acetabular remodeling and the persistence of dysplasia in patients with DDH. The study also examines outcomes related to residual acetabular dysplasia and avascular necrosis (AVN), with a focus on how each reduction method influences these factors. It is noted that open reduction may be a contributing risk factor for AVN, and the study aims to report the complications associated with each reduction type.

## Materials and methods

After obtaining the Institutional Review Board approval, a retrospective study was conducted at the authors' institution, reviewing records of all patients treated for hip dysplasia from 2016 to 2021. The cases excluded were those of children with hip dysplasia related to neuromuscular or syndromic conditions. In this study, data were collected from patients' medical records with idiopathic DDH who underwent closed or open reduction without additional surgical intervention and completed at least two years of follow-up. The study excluded patients with teratological, paralytic, and pathological dislocations. Additionally, patients who had previously received treatment (surgical or nonsurgical) at another facility, as well as those who underwent open reduction with pelvic osteotomy, were not included in the research.

The demographic data were recorded, and radiographic assessments were obtained at the following intervals: prereduction, immediately after reduction, and at six weeks, three months, six months, one year, two years, three years, and four years postreduction. All Children received postreduction treatment with spica casting for 12 weeks, with a cast trim to broomsticks at six weeks, followed by 1-2 months of abduction bracing after the cast was removed. Open reduction was indicated if stability couldn't be achieved with closed reduction or if a prior closed reduction had failed. All open reductions were performed using an anterior hip approach with capsulorrhaphy.

**Figure 1:**
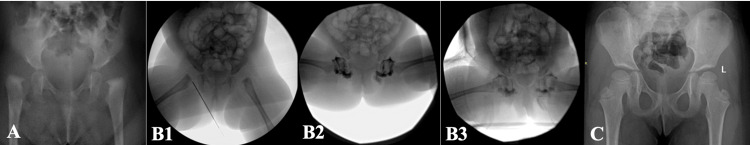
Anteroposterior pelvis radiographs of hip underwent closed reduction (A) preoperative radiograph, (B) intraoperative radiographs, (C) four years postoperative radiographs

**Figure 2:**
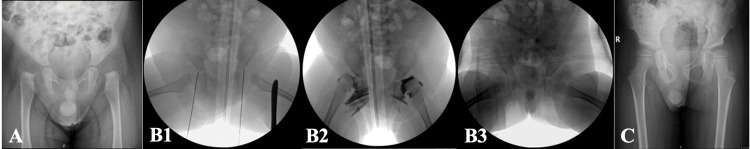
Anteroposterior pelvis radiographs of hip underwent open reduction (A) preoperative radiograph, (B) intraoperative radiographs, (C) four years postoperative radiographs

Anteroposterior (AP) radiographs were recorded at predetermined time points, and hips were classified according to the Tönnis classification. Acetabular index (AI) was measured throughout the follow-up period. AVN was graded according to Kalamchi and MacEwen on the outcome radiographs according to classification and reference (Figures 1, 2).

The patients were grouped according to the type of reduction into closed reduction group and open reduction group. The patients were further divided into two groups according to the type of reduction: closed or open. Residual dysplasia was defined as an AI greater than 30 degrees on the latest radiograph, applicable only to patients who completed four years of follow-up. The data were analyzed using IBM SPSS Statistics for Windows, Version 29 (Released 2023; IBM Corp., Armonk, New York, United States). For descriptive statistics, means (standard deviations) were reported for continuous variables and frequencies (percentages) for categorical variables. The comparison of groups was performed using the Student's t-test for continuous variables and the Chi-square test for categorical variables. Cramér’s V was used to assess effect sizes.

## Results

A total of 41 patients (64 hips) were included in this study. This group of patients included 31 female and 10 male patients. The mean age at reduction was 6.92 months (SD 7.18). The mean age at reduction was 6.92 + 7.18 months. The type of reduction was 38 (59.38%) closed reduction (Figure 1) and 26 (40.63%) open reduction (Figure 2). The mean follow-up duration was 51.5 + 15.07 months. The sex distribution included 10 males and 31 females. There were 28 (43.75%) right hips and 36 (56.25%) left hips (Table 1).

**Table 1:** Demographics

	Mean or frequency	% or SD
Age at reduction (months)	6.92	7.18
Follow up duration	51.5	15.07
Sex		
Male	10 (16 hips)	25.00%
Female	31 (48 hips)	75.00%
Hip		
Right	28	43.75%
Left	36	56.25%
Type of reduction		
Closed	38	59.38%
Open	26	40.63%

The association between categorical variables and the type of reduction was analyzed for all the hips. Among the patients who underwent closed reduction, 12 (31.58%) were males and 26 (68.42%) were females, while in the open reduction group, four (15.38%) were males and 22 (84.62%) were females (p = 0.142). For the hip side, 17 (44.74%) of the hips were right and 21 (55.26%) were left in the closed reduction group, compared to 11 (42.31%) right and 15 (57.69%) left in the open reduction group (p = 0.847). The presence of an ossific nucleus was noted in 21 (55.26%) of the closed reduction group and 14 (53.85%) in the open reduction group (p = 0.911). According to the Tönnis classification, none of the hips were classified as grade 1. Grade 2 was observed in eight (21.05%) hips in the closed reduction group and none of the open reduction group; grade 3 was observed in 18 (47.37%) hips in the closed reduction group and 10 (38.46%)in the open reduction group; and grade 4 was present in 12 (31.58%) hips in the closed reduction group and 16 (61.54%) of the open reduction group (p = 0.012). Residual dysplasia was noted in only one patient (4%) in the closed reduction group. AVN occurred in two (5.26%) hips in the closed reduction group and none in the open reduction group (p = 0.235) (Table 2).

**Table 2:** Association between the categorical variables and the type of reduction (no. of hips = 64) Results presented as n (%).  ^b^ measured in patients who completed four years of follow-up

	Closed reduction n = 38	Open reduction n = 26	χ^2^	Cramér’s V	p-value
Sex					
Male	12 (31.58)	4 (15.38)	2.16	0.18	0.142
Female	26 (68.42)	22 (84.62)			
Hip					
Right	17 (44.74)	11 (42.31)	0.04	0.02	0.847
Left	21 (55.26)	15 (57.69)			
Ossific nucleus	21 (55.26)	14 (53.85)	0.13	0.01	0.911
Grade (Tönnis classification)			8.92	0.37	0.012
1	0 (0)	0 (0)			
2	8 (21.05)	0 (0)			
3	18 (47.37)	10 (38.46)			
4	12 (31.58)	16 (61.54)			
Residual dysplasia^b^	1 (4)	0 (0)	0.94	0.11	0.482
Avascular necrosis	2 (5.26)	0 (0)	1.41	0.15	0.235

The association between the quantitative variables and the type of reduction was analyzed for 64 hips. The mean age at reduction was lower for hips treated with closed reduction (4.58 months, SD = 4.44) compared to those treated with open reduction (10.34 months, SD = 8.95), showing a statistically significant difference (p = 0.001). The change in the AI throughout the follow-up period didn’t show a statistically significant difference between the two groups (Table 3). 

**Table 3:** Association between the quantitative variables and the type of reduction (no. of hips = 64) Results presented as mean (SD)

	Closed reduction n = 38	Open reduction n = 26	p-value
Age at reduction (months)	4.58 (4.44)	10.34 (8.95)	0.001
Acetabular Index			
Pre- reduction (64 hips)	37.39 (6.52)	36.74 (4.41)	0.659
After 6 weeks (60 hips)	29.93 (7.49)	28.49 (6.97)	0.461
After 3 months (64 hips)	28.44 (5.95)	27.23 (5.56)	0.418
After 6 months (64 hips)	25.05 (4.52)	24.84 (5.41)	0.866
After 1 year (64 hips)	25.25 (3.58)	24.96 (4.97)	0.783
After 2 years (63 hips)	23.39 (3.69)	22.89 (5.11)	0.657
After 3 years (57 hips)	21.86 (3.72)	21.37 (4.7)	0.665
After 4 years (40 hips)	19.54 (4.56)	19.4 (5.06)	0.932
Acetabular Index change	17.32 (8.18)	16.83 (5.53)	0.792

## Discussion

The findings of this study demonstrated no significant difference in acetabular remodeling, as measured by the AI between open and closed reduction techniques. Additionally, while the rate of AVN was observed to be higher in the closed reduction group, the difference was not statistically significant. The majority (75%) of the participants included in the present study were females. This is consistent with the literature as the incidence of DDH in females is one in every 600 girls and one in every 3000 boys [14]. Furthermore, the involvement of the left was more common in the present study compared to the right hip. In approximately 63% of the cases, unilateral involvement has been reported in the literature and among those, 64% have left-sided involvement. This predominance can be attributed to the left occiput anterior position which is common in utero. In this position, the fetus’s left hip is adducted against the mother’s lumbosacral spine which leads to increased pressure and potential constraint on the left hip joint during development [15,16].

Age at the time of reduction is often considered a significant predictor of acetabular development. Previous studies have suggested that an earlier reduction may lead to better outcomes but the results are still inconsistent [17]. In the present study, we found a significant difference in the mean age at reduction between the closed and open reduction groups. The mean age in closed reduction was lower than in open reduction. Our findings contrast with a previous study by Abousamra et al. that reported no significant difference in age at reduction between open and close reduction [18]. The present findings can be explained by the fact that closed reduction is often attempted earlier and open reduction is usually considered when closed reduction fails.

The study evaluated the AI for four years. Although the AI showed improvement in both groups over time, there was no statistically significant difference between the two groups at any follow-up interval at six weeks, three months, six months, and at one, two, three, and four years. Similar to our results, there was no significant difference in outcome AI in closed and open reduction groups in the study by Abousamra et al. (25 ± 7vs. 22 ± 6 for closed and open reduction, respectively) [18]. Residual dysplasia, defined as an AI greater than 30 on the latest radiograph, was observed in only one case in the closed reduction group and none in the open reduction group. However, this difference was not statistically significant. Similar to our study, higher residual dysplasia was observed in closed reduction compared to open reduction (19% vs. 29%) in a previous study [18]. Morris et al., in their study, also reported that increased residual dysplasia was observed in closed reduction, and it was also related to increased risk of surgery [19]. Previous studies have reported that approximately 30% of closed reduction procedures require osteotomy and 49% require secondary surgery due to dysplasia in open reduction [20].

The Tönnis classification, which grades the severity of hip dislocation, revealed a higher association of severe dysplasia (grade 4) with the open reduction group (61.54%) compared to the closed reduction group (31.58%) (p = 0.012). Generally, open reduction is performed more in severe cases compared to closed reduction. In the present study, AVN was reported in two cases in a closed group, whereas no case was observed in open reduction. Our findings are not consistent with previous studies which have shown higher AVN in open reduction [21,22]. Abousamra et al. in their study also reported that AVN was higher with open reduction compared to closed reduction (23% vs. 7%) [18].

Limitations

The study had several limitations. As a retrospective study, it relied on existing medical records, which may sometimes be incomplete or inaccurate, potentially leading to bias. Additionally, this study was conducted at a single institution, which may limit the generalizability of the findings to other settings and populations. The relatively small sample size of 41 patients (64 hips) may affect the statistical power and the ability to detect significant differences between the groups. The follow-up duration, averaging 51.5 months, provides valuable medium-term data, but longer follow-up periods are necessary to fully assess long-term outcomes and complications, such as the development of osteoarthritis or the need for further surgical interventions. Radiographic assessments were subject to observer interpretation, potentially introducing observer bias despite efforts to standardize these measurements. The study did not include functional outcomes such as patient-reported hip function, quality of life, and long-term mobility, which are crucial for a comprehensive understanding of treatment success. Additionally, variations in surgical techniques, surgeon experience, and postoperative care practices could influence the outcomes, affecting the comparability of the study results. Future research should aim for larger, multicenter studies with extended follow-up periods and a focus on both radiographic and functional outcomes to enhance the generalizability and robustness of the findings.

## Conclusions

In conclusion, the findings of the present study showed that AI improved over the years in both groups, without any significant difference between the groups. The presence of residual dysplasia and AVN was higher in the closed reduction group compared to the open reduction; however, the difference was not statistically significant. The overall findings suggest that no significant difference between the two techniques in acetabular remodeling. Future studies should further investigate the findings of the present study in larger studies, with longer follow-up durations.
